# Effects of Age, Gender and Soil-Transmitted Helminth Infection on Prevalence of *Plasmodium* Infection among Population Living in Bata District, Equatorial Guinea

**DOI:** 10.3390/tropicalmed8030149

**Published:** 2023-02-27

**Authors:** Gertrudis R. Meñe, Maxmillian G. Mpina, Alejandro Lopelo, Elizabeth L. Nyakarungu, José Raso Bijeri, Antonio Martin Elo Elo, Florentino Abaga Ondo, Guillermo A. Garcia, Wonder P. Phiri, Ali Mohamed Ali, Jean Claude Dejon Agobé, Ayola Akim Adegnika, Salim M. Abdulla

**Affiliations:** 1 Faculty of Environmental Sciences, National University of Equatorial Guinea, Hassan II Avenue, Malabo P.O. Box 661, Equatorial Guinea; 2 Ecole Doctorale Régional de l’Afrique Centrale en Infectiologie Tropicale, Franceville 876, Gabon; 3 Centre de Recherches Médicales Lambaréné, Lambaréné 242, Gabon; 4 Laboratorio de Investigación de Baney, Baney District Hospital, Baney, Equatorial Guinea; 5 MCD Global Health, 8403 Colesville Rd, Suite 320, Silver Spring, MD 20910, USA; 6 Ifakara Health Institute, Dar-es-Salaam P.O. Box 78373, Tanzania; 7 Swiss Tropical and Public Health Institute, Kreuzstrasse 2, Allschwil, 4123 Basel, Switzerland; 8 Equatorial Guinea Ministry of Health, Calle del Rey Malabo, Malabo, Equatorial Guinea; 9 Institut für Tropenmedizin, Universität Tübingen and German Center for Infection Research, Wilhelmstraße 27, 72074 Tübingen, Germany

**Keywords:** malaria, soil-transmitted helminth, prevalence

## Abstract

Introduction: Malaria and soil-transmitted helminth (STH) co-infection is an important parasitic infection affecting populations in co-endemic countries including Equatorial Guinea. To date, the health impact of STH and malaria co-infection is inconclusive. The current study aimed to report the malaria and STH infection epidemiology in the continental region of Equatorial Guinea. Methods: We performed a cross-sectional study between October 2020 and January 2021 in the Bata district of Equatorial Guinea. Participants aged 1–9 years, 10–17 years and above 18 were recruited. Fresh venous blood was collected for malaria testing via mRDTs and light microscopy. Stool specimens were collected, and the Kato–Katz technique was used to detect the presence of *Ascaris lumbricoides*, *Trichuris trichiura*, *hookworm* spp. and intestinal Schistosoma eggs. Results: A total of 402 participants were included in this study. An amount of 44.3% of them lived in urban areas, and only 51.9% of them reported having bed nets. Malaria infections were detected in 34.8% of the participants, while 50% of malaria infections were reported in children aged 10–17 years. Females had a lower prevalence of malaria (28.8%) compared with males (41.7%). Children of 1–9 years carried more gametocytes compared with other age groups. An amount of 49.3% of the participants infected with *T. trichiura* had malaria parasites compared with those infected with *A. lumbricoides* (39.6%) or both (46.8%). Conclusions: The overlapping problem of STH and malaria is neglected in Bata. The current study forces the government and other stakeholders involved in the fight against malaria and STH to consider a combined control program strategy for both parasitic infections in Equatorial Guinea.

## 1. Introduction

Malaria continues to be a disease of public health importance despite the tremendous efforts invested in the control of *Plasmodium* spp. in endemic countries [1,2]. The emergence of the COVID-19 pandemic at the beginning of 2020 has complicated the effort against infectious diseases in most African countries where public health services were facing serious challenges [2]. There has been globally substantial progress in malaria reduction in Africa. However, in some parts of the African continent, the epidemiology of malaria infection has remained unchanged or has increased in some pockets, calling for an urgent investigation and action for strengthening its control [3]. In 2021, the World Health Organization (WHO) malaria report estimated over 240 million malaria cases and over 620,000 malaria deaths worldwide [2]. These data represent an increment of about 14 million cases and 69,000 deaths compared with the year 2020. The report further suggested that the increment of 10 cases of incidence per population of 1000 at risk in 2020 was attributed to impaired health systems due to the COVID-19 pandemic [2].

Equatorial Guinea (EG) is a Central African country and constitutes two regions: the insular and the continental regions. The continental region comprises a large part of the country’s surface area, while the insular region is characterized by two islands, namely, Bioko and Annobón. The country is holoendemic for *Plasmodium* infection with a year-round malaria transmission pattern [4]. A malaria control program initiated in 2004 by MCD Global Health (previously Medical Care Development International (MCDI)) on Bioko Island significantly contributed to reducing malaria cases, although such improvement has not shown a clear reduction in malaria cases in the island to date [5,6,7]. In 2007, a similar malaria interventional model (an extensive program of indoor insecticide spraying, long-lasting insecticidal net distribution, expanding access to diagnostics and treatment, and surveillance) was adapted to the continental region of Bata. However, since EG was no longer able to obtain funds from the Global Fund, the inability to sufficiently scale up the coverage of intervention in Bata led to poor outcomes [6]. Recently, the burden of *Plasmodium falciparum* (*P. falciparum*) malaria in EG has been reported to be high in the continental region of Bata compared with Bioko Island, which is most likely due to ongoing intensive and successful vector control programs that are ongoing on Bioko Island [7,8,9,10].

The combination of malaria control strategies, such as malaria vaccines, vector control, and improved diagnosis and treatment, could be crucial for sustainable disease control [11,12,13]. A clear understanding of the patterns of the disease epidemiology can additionally provide important information to support the prioritization of malaria control strategies [14,15,16]. Disease prevalence represents one of the traditional and easy-to-implement assessment tools for the measurement of disease status in an area. Several studies have reported prevalence data from Bioko Island where the malaria control program is firm [6,13,17,18], and some suggested that a large proportion of cases seen in Bioko Island are imported from the Bata district [8,19,20]. However, sufficient data on the prevalence of malaria from the continental region are scarce, and the latest prevalence data from the continental region, especially in the Bata district, were reported in 2015 [21,22].

Age and sex are among the most studied factors that have been shown to influence different disease outcomes. Several studies have reported that malaria prevalence is affected by age [23] or age-dependent sex bias [24], whereas others show the contradicting influence of sex on malaria prevalence in different locations [21]. While studies conducted in Nigeria [25] and Bioko Island, EG [10] have demonstrated that males are more at risk of acquiring malaria infection, the studies conducted in Uganda showed that females are more vulnerable to acquiring malaria infection [26]. However, the effects of age, sex and the type of STH infection on malaria have not been well extrapolated.

Soil-transmitted helminths (STHs) refer to intestinal worms transmitted to humans through contaminated soil, causing substantial disease and disability to more than a quarter of the world’s population [27,28]. STHs live in the intestines, where they produce thousands of eggs a day that can be transmitted to another person or contaminate the soil in areas where sanitation is poor [27]. The health impact of STH infection is directly related to worm burden, as light STH infection usually has no symptoms, while heavy infection contributes to anemia, malnutrition, growth stunting and low birth weight [29]. Moderate to heavy STH infection also leads to impairment of physical and mental growth, delayed educational advancement and a negative impact on economic development [29]. The prevalence data on STH in EG are not well established due to numerous deficiencies that include a lack of programs and research that focus on neglected tropical diseases including STH and a scarcity of health education and sensitization [30]. The most common STHs in Central Africa including EG, amongst others, include *Ascaris lumbricoides (A. lumbricoides), Trichuris trichiura (T. trichiura), Necator americanus (N. americanus)* and *Ancylostoma duodenale (A. duodenale)* [31]. While successful prevention of STH requires extensive preventative chemotherapy and a public health communication plan, improvements in community living and sanitation standards have clearly contributed to the elimination of STH in many countries that have successfully eliminated STH infection [32]. Such intervention is lacking or insufficiently implemented in EG and has possibly contributed to the sustainability of STH infection in a greater proportion of communities in the country.

Furthermore, malaria and helminth co-infection is the most common predominant infection affecting populations in Central Africa including EG due to their overlapping disease geographical distributions [33]. To date, the health impact of STH co-infection with malaria is inconclusive. A number of scientific reports have demonstrated a positive association between STH and malaria [34,35], and some studies showed that having STH increases susceptibility to severe malaria [36,37], while others reported a neutral association of STH–malaria co-infection with individual health [34,38,39]. Therefore, an overview of how the presence of STH influences the acquisition of malaria or its severity will add value in strategizing malaria control programs. The current study aimed to report updated and the most recent malaria prevalence data and parasite density distribution by age, sex and type of STH infection from the continental region of EG.

## 2. Materials and Methods

### 2.1. Study Design

The cross-sectional survey study took place in the two municipalities of the Bata district in the continental region of EG, selected using multi-stage cluster sampling. First, one district was randomly (simple random) selected, and within the district, two municipalities were selected. Second, from each municipality, four communities were selected using simple random sampling. Thereafter, 26 households were selected using systematic random sampling from each community based on a computer-generated random number list applied to the community register of households. Lastly, two individuals were selected from each household using stratified random sampling. A randomized list of the age categories for each household was produced. In cases where more than one person in the selected house was within the same age group, participants were chosen by tossing a coin. With the above sampling, 406 individuals were selected to participate in this study.

### 2.2. Study Area

This study was conducted in two municipalities of the Bata district (Bata and Rio campo) in the continental region of EG between October 2020 and January 2021. These municipalities represent the urban (Bata) and rural (Rio Campo) settings of the most populated district in the mainland (the Bata district). The sites were selected based on their population sizes as well as based on the convenience to perform this study.

### 2.3. Study Population

Volunteers were equally categorized into 3 age groups—pre-school children (1–9 years), school-age children (10–17 years), and above 18 years—and were recruited from the two municipalities of the Bata district, Continental Region of EG. The participants who resided in the study area for at least 3 months before the survey, provided signed or thumb-printed and witnessed informed consent/permission/assent, and met the above age criteria were included in this study. Participants with no thick blood smear (TBS) results were excluded from the analysis.

### 2.4. Sample Size Calculation

The sample size was calculated based on the ability to estimate a prevalence of malaria of approximately 50% with a margin of error of approximately 5%; hence, a minimum of 400 volunteers needed to be surveyed (including 5% of the volunteers who were not able to provide a blood specimen for this study).

### 2.5. Laboratory Analysis

#### 2.5.1. Malaria Diagnosis

The commercially available RDT (Carestart malaria Pf/PAN (PfHRP2/pLDH) Ag Combo, ACCESSBIO, USA (Lot# MR19L62, expiration: 30 April 2022)) was used throughout the participant recruitment process in the field, according to the manufacturer’s instructions and as detailed elsewhere [40]. Two milliliters of fresh venous blood was collected from participants for malaria testing with malaria rapid diagnostic tests (mRDTs) in the field and sent to the lab for the confirmation and identification of *Plasmodium* spp. using optical light microscopy. The thick blood smears (TBSs) were prepared by evenly spreading 10 µL of fresh venous blood on a 1 cm × 2 cm rectangle for a thick smear and 6 µL of blood to obtain a thin smear [41]. The smears were air dried, stained for 45 min using a 4% Giemsa stain, and rinsed with buffered water (pH 7.2). The slides were dried and read using a light microscope with a high-power field (immersion oil, 100× objective) of a 0.18 mm diameter. Passes of 6 × 1 cm equivalent to 0.54 µL of blood or 24 × 1 cm passes equivalent to 2.14 µL of blood for symptomatic volunteers were read before TBSs were declared positive or negative. A TBS was considered positive when two independent expert microscopists reported two or more clearly defined parasites and considered negative when no parasite was reported. A third microscopist resolved any discrepancy between the two readers. For Giemsa stain quality control, known positive and negative thin blood smears were included at the beginning of the day and analyzed for both parasites and cell staining color and quality according to a standard operating procedure. Only Giemsa stains that passed the quality control procedures were allowed to be used for the slide staining on that day. Microscopes were maintained on a daily basis [40]. The mRDT results that were confirmed with TBS were used for analysis. Since mRDT has been routinely used for malaria diagnosis across the country, participants who had mRDT-positive results were treated according to the national guidelines for malaria treatment.

#### 2.5.2. Kato–Katz Technique for Helminth Diagnosis

The Kato–Katz technique was used for the diagnosis of *A. lumbricoides, T. trichiura*, *hookworm* spp. and *Schistosoma* spp., as described elsewhere [42]. Briefly, glass slides were labeled with the sample identification, and plastic templates of 41.7 mg were placed on top of the labeled slides. Approximately 50 mg of fecal material was placed on a newspaper or glazed tile, and a piece of nylon screen was pressed on top. Using a spatula, the sieved fecal material was scraped through the screen to fill the hole in each of the templates, leveling the feces off to remove any excess. Carefully, the templates were lifted off and one of the cellophanes, which had been soaked overnight in methylene blue glycerol solution, was placed over each of the fecal samples. The microscope slides were inverted and the fecal samples were firmly pressed against the cellophane strips on a smooth tile surface. The material was spread evenly. Carefully, the slides were removed by gently sliding them sideways to avoid separating the cellophane strips, placed upwards and let stand at room temperature for 1 h. The smears were examined using microscopy in a systematic manner by two independent technicians, and the eggs of each species were reported.

### 2.6. Statistical Analysis

Data were collected and entered into Microsoft Excel^®^ (Microsoft Corporation, 2016). Descriptive statistics were used to summarize the data. For categorical data frequency, counts and percentages were used, and continuous variables were summarized with means, standard deviation and ranges. The associations between categorical variables were tested using Chi-squared tests. In the univariate logistic regression, a variable that showed association at a 20% significance level was considered in the multivariate analysis. Backward elimination with the use of a likelihood ratio test at a significance level of 5% was used to select the final model. A *p*-value of 0.05 or less was considered statistically significant unless stated otherwise. The data analysis was performed with STATA 15 standard editions (STATA Corp, College Station, TX, USA).

### 2.7. Ethical Considerations

The protocol received approval from the EG National Ethics Committee (CENGE; Nr Reg-2019-028). Furthermore, this study was also approved by the public health direction of the Ministry of Health and Social Welfare (reference number 56-150), the regional delegation of Bata of the Ministry of Health and Social Welfare (reference number 2018), and the government delegation of Bata, Littoral from the Ministry of Interiors and Local Corporations (reference number 937). In addition, the protocol was submitted and approved by the training host institution, the Centre de Recherches Médicales de Lambaréné (CERMEL) for scientific and technical advice. All study procedures were conducted according to Good Clinical Practice (GCP). All eligible participants were asked to provide their consent by signing an informed consent form before enrollment in this study.

## 3. Results

### 3.1. Study Participants Demographic Characteristics

A total of 406 participants were screened, 4 [4] of them were withdrawn before signing the consent form and only 402 were included in the analysis. A total of 63 of the 402 participants could not provide fecal specimens during the screening. A total of 22 of the 63 participants who could not provide fecal specimens were malaria positive and 41 out of 63 were malaria negative. The mean (range) age was 24 (1.0 to 86.0) years, and 53.5% of participants were females. A total of 178 (44.3%) of the volunteers were living in urban areas. Of the 160 adults (above 18 years) interviewed on having a bed net, only 83 (51.9%) of them reported having bed nets. A summary of the baseline demographic characteristics of the study participants is shown in Table 1.

### 3.2. Distribution of Malaria and STH Infection

**Figure 1:**
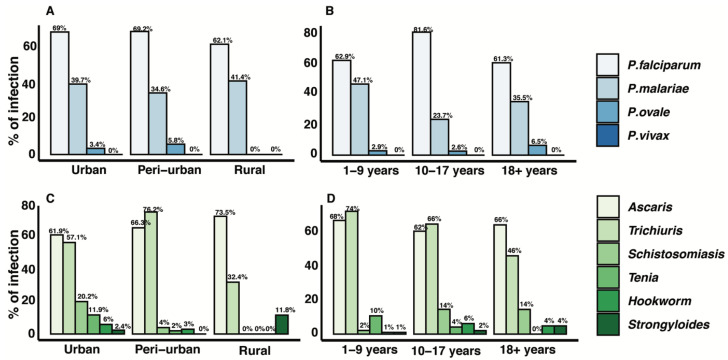
Distribution of malaria infection (**top** row) and helminth (**bottom** row). Panel **A** is the distribution of plasmodium by area and panel **B** is by age. Panel **C** is the distribution of helminth by area and panel **D** is by age.

The distribution of infection by area and age is presented in Figure 1. In all areas, *P. falciparum* was the most frequent species identified, followed by *P. malariae* (Figure 1, panel A). This trend was also observed in all age groups. Participants aged 10–17 years had the highest proportion of *P. falciparum* compared with other age groups. Participants aged 1–9 years also had a higher prevalence of *P. malariae,* while *P. ovale* was prevalent in participants aged 18 years and above. *P vivax* was not detected in our sampled population (Figure 1, panel B). For helminth, *A. lumbricoides* was most prevalent in urban and rural areas, while *T. trichiura* was highest in peri-urban areas. A higher prevalence of *T. trichiura* was also observed in urban and rural areas. More than 66% of *A. lumbricoides* was observed in peri-urban areas (Figure 1, panel C). Participants aged 1–9 years have the highest prevalence of *A. lumbricoides* and *T. trichiura* compared with other age groups. In addition, although *Taenia* spp. was not a major focus in the current study, *Taenia solium (T. solium)* was detected during the analysis and was most frequent in the 1–9 years age group (Figure 1, panel D).

### 3.3. Prevalence of Malaria Infection and Age

The current study showed that 140 (34.8%) participants had malaria-positive results via mRDT that were confirmed using TBS. The prevalence of malaria was higher (50%) in children aged 10–17 years compared with those of 1–9 years (42.2%) and above 18 years (20%). The prevalence of malaria was lower in females (28.8%) compared with males (41.7%), as shown in Table 2.

**Figure 2:**
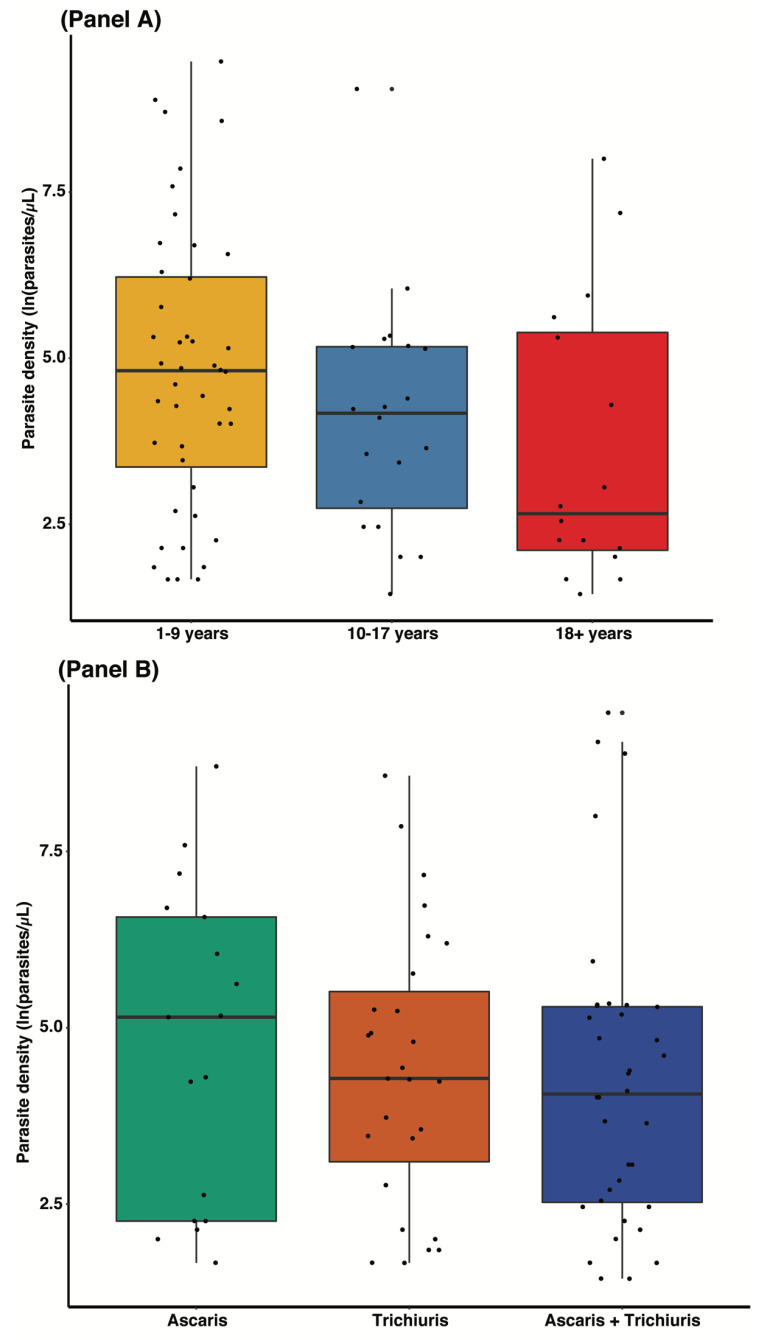
Distribution of parasite density by age group (**Panel A**) and by type of soil—transmitted helminths (**Panel B**).

**Figure 3:**
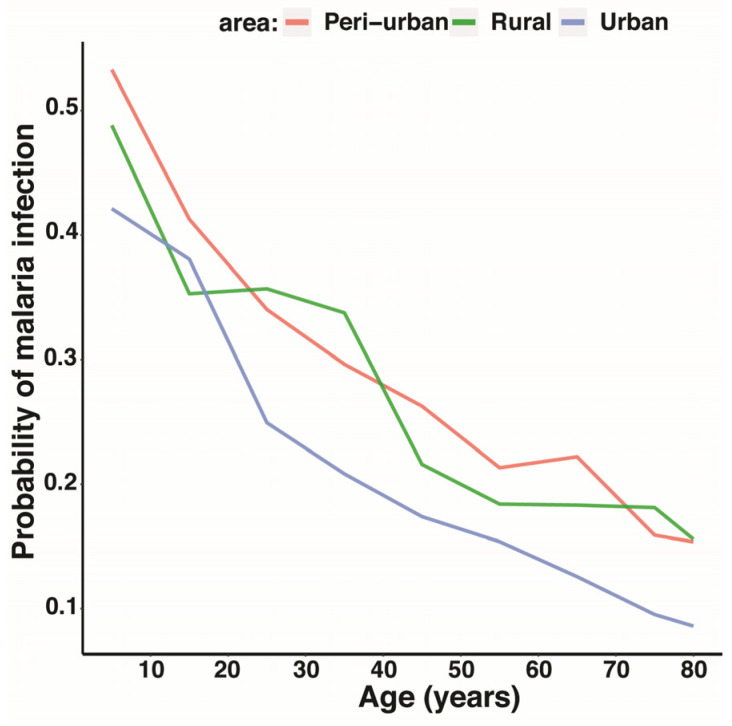
Predicted probability of malaria infection from the final logistic regression model.

The results of the univariate logistics regression indicate that the risk of malaria infection decreased with age (Figure 2A and Figure 3), whereby each year an increase in age was associated with a 2% decrease in the risk of malaria. Male participants have 77% more risk of being infected with malaria compared with females (Table 2). Children of 1–9 years carried more gametocytes compared with other age categories, and participants living in peri-urban areas had more gametocytes than those living in urban or rural areas (Table 3). The prevalence of malaria in participants using LLINs was 18.1% compared with 39.2% in participants who do not use LLINs; however, the difference in the odds of malaria infection between these 2 groups was not statistically significant (odds ratio, [95% CI]: 0.78 [0.36–1.69] (Table 2)).

In the multivariate logistics regression, only the differences amongst ages (as continuous) and sex remained significant. The results of the final model are presented in Table 2. Each year, an increase in age was associated with a 2% decrease in the risk of malaria, given that other factors were adjusted. After adjusting for age and area, the risk of malaria was 60% higher in males compared with females. After adjusting for age and sex, living outside urban areas was associated with more than 30% higher risk of malaria infection, although the observed difference was not statistically significant (*p*-value > 0.05).

### 3.4. Prevalence of Malaria Infection and STH Infections

Figure 2 shows the distribution of malaria parasite density by age and helminth species. In general, participants who were infected with *A. lumbricoides* had more malaria parasites compared with those who were infected with *T. trichiura* or both (Figure 2, panel B). Furthermore, the current study reported the prevalence of malaria to be significantly higher (40.6%) in participants who were co-infected with STH (Table 4), although we did not find any association between parasite density and the type of STH (Figure 2, panel B). Although other helminths species were not a major focus of the current study, *Schistosoma intercalatum (S. intercalatum), Ancylostoma* spp., *Taenia solium* and *Strongyloides stercoralis* mono, double and triple infection were detected during the analysis (Table 4). The current study showed a negative trend association (not statistically significant) between malaria and schistosomiasis (Figure 1). The data showed that 76.2 % of malaria-negative individuals had *Schistosoma intercalatum* infection compared with 23.8% of those who were malaria-positive.

## 4. Discussion

The current study aimed to determine the burden of malaria infections and associated risk factors in the Bata district of EG. For decades, malaria remained as a disease of public health importance and continued to be a leading cause of morbidity and mortality in EG. This study compared its prevalence by gender, and, herein, we demonstrated that males had a higher prevalence of malaria infection (41.7%) compared with females (28.8%), affirming the finding of previous studies conducted in similar settings in Nigeria and Cameroon, and in Malaysia [25,43,44]. This could be attributed to socio-economic activities that exposed males to outdoor activities, hence exposing them to more mosquito bites at night [44]. In addition to this, males tend to not attend health facilities when they are ill, and this may lead to underestimates of the current outcomes for males in the health facilities databases, hence affecting prevalence studies that are based on hospital data [45].

On the contrary, a number of studies have also reported higher malaria prevalence in females compared with males [26,45,46], the reasons being that females tend to stay outside for longer at night selling commodities and seek medical care from health facilities more often compared with their male counterparts, regardless of symptoms of malaria. This might lead to malaria being recorded more in the female group. However, carefully conducted clinical studies did not suggest the existence of a significant difference in malaria prevalence between genders [45].

When the current study compared the prevalence by age groups, the data showed that older children and early teenagers (10–17 years) had a higher prevalence of malaria (50%) followed by toddlers (1–9 years) (42.2%), while adults above 18 years had the least prevalence (20%). The analysis suggests that the higher malaria prevalence reported in the 10–17 years age group could be attributed to their active habits of staying outside houses during early nights, which increases exposure to mosquito bites, or/and the delayed acquisition of natural immune responses due to interventions implemented at a young age [47]. Similar findings were reported by previous studies conducted in Mali [48] and in South-East Nigeria [49]. Furthermore, when comparing malaria prevalence by area of residence, we showed that living in rural and peri-urban areas was associated with a higher risk of malaria, although this increase was not statistically significant.

Next, there is a need to investigate the distributions of parasite density among age groups. Herein, the data show that malaria parasite density declines as individuals increase in age. This study also showed that the risk of malaria decreased with age, whereby each year an increase in age was associated with a 2% decrease in the risk of malaria. The findings of the current study are in line with several studies that have also reported similar findings [50,51,52] and could reflect the immunological evidence that shows that malaria immunity, which can also reduce the parasite multiplication rate, is acquired as individuals age in malaria-endemic settings; however, there is no clear concept about how this protection works [53,54].

Gametocytes are drivers of malaria infection as they determine the transmission of malaria from one individual to another via *Anopheles mosquitos* [55]. Therefore, there remains a need to investigate the distribution of gametocytes amongst the study areas and age groups. Herein, this study showed that children of ages between 1 to 9 years carry a higher number of gametocytes compared with other age group categories. Similar findings were reported in a study conducted in Mali by Adomako-Ankomah et al. [56,57]. This finding could be explained by the higher amount of parasite density demonstrated in this age group. Furthermore, people who live in peri-urban areas were found to harbor more gametocytes compared with those who live in urban and rural areas. The findings of this study also support the results of a study conducted in Latin America [58].

Bed nets (long-lasting insecticidal nets (LLINs)) have been the most successful malaria intervention to date [59]. Herein, this study further demonstrated that the prevalence of malaria in participants who reported using bed nets was 18.1% compared with 22.1% in participants who did not use bed nets. However, this study did not find any statistically significant difference in the odds of malaria infection between people who reported using bed nets and those who did not report using LLINs (odds ratio, [95% CI]: 0.78 [0.36–1.69]). Contrary to the findings of the current study, previous studies have shown that the use of LLINs is associated with a significant reduction in mortality, uncomplicated malaria incidence and parasite prevalence in children [60]. Sustaining a high coverage of LLINs together with other interventions has been proven to lower the burden of the disease and reach the long-term goal of malaria elimination [61]. The current study reported that only 20% of the study participants were using LLINs. The coverage reported in the current study was low and might have supported the high case prevalence recorded in the current study. The national malaria control programs, which are the pioneers of the communicating components of effective LLIN distribution and behavior change, should ensure that the population uses LLINs consistently.

The body of evidence suggests that soil-transmitted helminths (STHs) have some modulatory effect on the pathophysiology of malaria infection [62,63]. Therefore, this study further investigated the prevalence of malaria in a group of individuals categorized with and without STH. The analysis showed that the prevalence of malaria infection was significantly higher (40.6%) in participants who were co-infected with STH compared with those without STH infection. When comparing the prevalence of malaria by the type of STH infection, we showed that malaria prevalence was higher in participants who were mono-infected with *T.trichiuras* (49.3%), followed by those who were multiply infected with both *A. lumbricoides* and *T. trichiuras* (46.8%). Moreover, when comparing the parasite densities amongst participants infected with different types of STH, we found that those who had malaria co-infected with *A. lumbricoides* had a slightly higher median density compared with *T. trichiuras* alone or *T. trichiuras* and *A. lumbricoides* mixed infections, although the evidence of association did not reach a level of statistical significance. Contrary to the findings of the current study, a positive association between STH and *Plasmodium* parasite density has been reported elsewhere [36].

The current study had some limitations. Amongst these were a lack of qPCR data for the malaria parasite, HRP2 gene deletion and STH diagnosis. This might have led to an underestimation due to the use of TBS and mRDT, which are not as sensitive as qPCR, or an overestimation of the cases due to false positive reporting. On the other hand, the inclusion of individuals who had stayed in the study areas for at least 3 months might also have led to an overestimation of prevalence, as individuals may come from other areas with infection. The current study also used a single fecal sample collection to estimate STH infection. This may have contributed to an underestimation of STH diagnosis, especially when the densities of some species were low. However, the use of a single sampling time point from cross-sectional studies may be sufficient to obtain a close estimate of current STH infection. Furthermore, there were no data on malaria prevalence just before COVID-19 started, hence hindering a proper comparison within the current study.

## 5. Conclusions

This study demonstrated that despite global interventions against malaria, cases of malaria have plateaued in Bata, the continental region of EG. This study reinforces the importance of the government and other stakeholders involved in the fight against malaria in EG re-thinking new interventions. The data from the current study suggest that the risk of contracting malaria and STH is high in EG; hence, a combined strategy to fight both protozoa and helminth infection will benefit the interventions for parasitic infection in EG. However, further studies covering more areas of the continental region and with more sensitive diagnostics methods will supplement the data provided by the current study.

## Figures and Tables

**Table 1:** Demographic characteristics of the study participants.

Parameter	Age Group			All
	1–9 Years	10–17 Years	≥18 Years	
Sex				
Female	76 (45.8)	36 (47.4)	103 (64.4)	215 (53.5)
Male	90 (54.2)	40 (52.6)	57 (35.6)	187 (46.5)
Mean age ± SD (range) years	5.3 ± 2.5 (1.0, 9.8)	12.4 ± 2.0 (10, 17.5)	49.0 ± 16.8 (18.2, 86.1)	24.0 ± 23.1 (1.0, 86.1)
LLINs				
Having LLINs	NA	NA	83 (51.9)	
Area				
Urban	72 (43.4)	54 (71.1)	52 (32.5)	178 (44.3)
Peri-urban	63 (37.9)	16 (21.0)	55 (34.4)	134 (33.3)
Rural	31 (18.7)	6 (7.9)	53 (33.1)	90 (22.4)

LLINs = long-lasting insecticidal nets.

**Table 2:** Final logistic regression model.

	Malaria Positive (%)	Univariate		Multivariate		
Parameter		Odds Ratio (SE)	95% CI	Odds Ratio (SE)	95% CI	*p*-Value
Age groups (years)						
Age (year)		0.98 (0.01)	0.97, 0.99	0.98 (0.01)	0.97, 0.99	<0.001
1–9	42.2	Reference				
10–17	50	1.37 (0.38)	0.79, 2.36	-	-	-
≥18	20	0.34 (0.09)	0.21, 0.56	-	-	-
Sex						
Female	28.8	Reference		Reference		
Male	41.7	1.77 (0.37)	1.17, 2.67	1.6 (0.35)	1.04, 2.46	0.031
LLINs						
No LLINs	39.2	Reference		Reference		
With LLINs	18.1	0.78 (0.31)	0.36, 1.69	-	-	-
Area						
Urban	32.6	Reference		Reference		
Peri-urban	39.6	1.35 (0.32)	0.85, 2.16	1.6 (0.4)	0.98, 2.6	0.059
Rural	32.2	0.98 (0.27)	0.57, 1.69	1.33 (0.39)	0.75, 2.35	0.335

LLINs = long-lasting insecticidal nets.

**Table 3:** Distribution of gametocytes amongst the study participants by age and area category.

Parameter	Number of Gametocytes (Gametocyte/µL of Blood)		
	0	1–4	>5
Age (years)			
1–9	84 (79.3)	14 (13.2)	8 (7.6)
10–17	39 (79.6)	8 (16.3)	2 (4.1)
≥18	38 (77.6)	8 (16.3)	3 (6.1)
Area			
Urban	59 (81.9)	8 (11.1)	5 (6.9)
Peri-urban	65 (74.7)	15 (17.2)	7 (8.1)
Rural	37 (82.2)	7 (15.6)	1 (2.2)

**Table 4:** Association between malaria and soil-transmitted helminths.

Soil-Transmitted Helminths	Malaria	
	Negative (%)	Positive (%)
Negative	91 (75.8)	29 (24.2)
Positive	130 (59.4)	89 (40.6)
Mono-infection		
*Ascaris lumbricoides*	37 (68.5)	17 (31.5)
*Ancylostoma* spp.	2 (66.7)	1 (33.3)
*Schistosoma intercalatum*	8 (80)	2 (20)
*Strongyloides stercoralis*	2 (66.7)	1 (33.3)
*Trichiuris trichiuras*	24 (49)	25 (51)
*Taenia solium*	3 (100)	0 (0)
Double infections		
*Ascaris lumbricoides + Ancylostoma* spp.	1 (100)	0
*Ascaris lumbricoides + Schistosoma intercalatum*	2 (66.7)	1 (33.3)
*Ascaris lumbricoides + Strongyloides stercoralis*	1 (100)	0 (0)
*Ascaris lumbricoides + Trichiuris trichiuras*	34 (48.6)	36 (51.4)
*Ascaris lumbricoides + Taenia solium*	3 (100)	0 (0)
*Ancylostoma* spp. + *Schistosoma intercalatum*	1 (100)	0 (0)
*Schistosoma intercalatum + Taenia solium*	1 (100)	0 (0)
*Schistosoma intercalatum + Trichiuris trichiuras*	1 (33.3)	2 (66.7)
*Strongyloides stercoralis + Trichiuris trichiuras*	0 (0)	1 (100)
*Tenia solium + Trichiuris trichiuras*	1 (100)	0 (0)
Triple infections		
*Ascaris lumbricoides + Ancylostoma* spp. + *Trichiuris trichiuras*	2 (50)	2 (50)
*Ascaris lumbricoides + Schistosoma intercalatum + Trichiuris trichiuras*	3 (100)	0 (0)
*Ascaris lumbricoides + Strongyloides* spp. + *Trichiuris trichiuras*	0 (0)	1 (100)
*Ascaris lumbricoides + Taenia solium + Trichiuris trichiuras*	4 (100)	0 (0)

## Data Availability

The data presented in this study are available upon special request.
